# The measurement of entrance surface dose using optically stimulated luminescence dosimeters for determining average glandular dose in digital breast tomosynthesis: Measurement and simulation study

**DOI:** 10.1002/acm2.13485

**Published:** 2021-11-29

**Authors:** Thunyarat Chusin, Sumalee Yabsantia, Kosuke Matsubara

**Affiliations:** ^1^ Department of Radiological Technology, Faculty of Allied Health Sciences Naresuan University Muang District Phitsanulok Thailand; ^2^ Department of Quantum Medical Technology, Faculty of Health Sciences, Institute of Medical, Pharmaceutical and Health Sciences Kanazawa University Kanazawa Ishikawa Japan

**Keywords:** average glandular dose, mammography, Monte Carlo simulation, nanoDot

## Abstract

This study aimed to evaluate the feasibility of using optically stimulated luminescence dosimeters (OSLDs), nanoDots, for the determination of an average glandular dose (AGD) with a specific digital breast tomosynthesis (DBT) system, whereas the X‐ray tube was fixed (2D mode) and moved (3D mode). The entrance surface air kerma (ESAK) was measured by placing the nanoDots on the surface of a polymethyl methacrylate (PMMA) phantom with 25, 28, and 34 kV W/Rh techniques. The experimental setup of the ESAK measurement was simulated using a Monte Carlo simulation code to determine the ESAK and the backscatter factor (BSF). The AGD was calculated by dividing the ESAK values over the corresponding BSF factors for each PMMA phantom thickness and multiplying the AGD conversion factors. The AGD determination by the nanoDots variated within ±5% for both 2D and 3D modes, compared to those determined using an ionization chamber. The results were similarly observed for the simulation, except for the 25 kV on the 3D mode. Regarding the International Atomic Energy Agency technical reports series number 457, the nanoDots can be used for the AGD determination with realistic 2D and 3D image acquisitions based on ±10% uncertainty.

## 1 INTRODUCTION

A meta‐analysis study showed the high‐potential use of digital breast tomosynthesis (DBT) for breast cancer screening with more sensitivity and specificity than using full‐field digital mammography (FFDM) alone.^1^ DBT is a 3D imaging technique that combines the use of tomography and 3D image reconstruction to improve lesion visibility.^2^ The DBT system such as the Siemens MAMMOMAT Inspiration system has both 2D and 3D image acquisitions.^3^ The X‐ray tube rotates and fires short X‐ray pulses over an angular range of 50° across a compressed breast to acquire a 3D image, whereas for a 2D image, it remains fixed. The average glandular dose (AGD) received by the glandular tissue is used as a gold standard for dosimetry in mammography. According to the protocol for the quality control of the physical and technical aspects of DBT systems,^4^ AGD is calculated using the following formula:

(1) AGD=K×g×c×s×T,

where *K* is the incident air kerma at the upper surface of the breast measured when the X‐ray tube pauses at the zero‐degree position, because an angular response of a standard dosimeter is taken into account. The *g*, *c*, *s*, and *T* factors were provided by Dance et al.,^5^, ^6^, ^7^, ^8^ and *g* is the air kerma to AGD conversion factor for the breast with 50% glandularity, *c* is a factor for different breast glandularity other than 50%, *s* is a factor for different target/filter combination from Mo/Mo, and *T* is an added factor to correct the AGD for X‐ray tube position with nonzero degree regarding the 3D image acquisition. However, the fixed X‐ray tube with 3D acquisition mode is not used in a clinical scenario. In addition, a *T* factor is needed to be investigated for a new DBT technology with a lack of information in the recent AGD conversion factor database.

Currently, an optically stimulated luminescence dosimeter (OSLD) has become more widely used in diagnostic radiology for experimental and clinical dose measurements. Because OSLD has less complications of annealing and reading processes, this makes it easier to use in clinical dose measurements. The measured dose can be read out multiple times for OSLD with less than 1.2% signal depletion in which thermoluminescent dosimeter (TLD) could not be found.^9^ An accumulated dose measurement with multiple exposures using OSLD could be applied at a low dose level, such as entrance surface dose (ESD) for mammography,^10^ with a reliability response of ±0.6% compared to a single exposure. The study by Kawaguchi et al.^12^ showed that the energy dependence of the OSLD; Al_2_O_3_:C (nanoDot) was less than 5.0% for the mammography energy range of 24–35 kV, which was consistent with Alothmany et al.^11^ who also reported the energy dependence was less than 5.0% in the 25–32 kV range. However, Kawaguchi et al.^12^ reported that the angular dependence of the nanoDots was lower than 4.0% for the X‐ray tube rotated in the range of ±30°, whereas those of 10.0% for Alothmany et al. Rocha et al.^13^ used the nanoDots and PTW QC dosimeter to measure the radiation dose for FFDM with 27 kV Mo/Mo target/filter combination. They found discrepancies between the nanoDots and PTW QC dosimeter of 0.5% and 0.6% in ESD and AGD, respectively. It has been suggested that nanoDots may be used as an alternative dosimeter for determining AGD in FFDM with reliable results. To the best of our knowledge, no study has been reported the use of OSLD to determine AGD in a realistic 3D image acquisition. Therefore, an investigation is needed to clarify the validity and reliability of using the nanoDots as an alternative dosimeter in 3D image acquisition with a clinical scenario.

The aim of this study was to validate the use of OSLD for measuring radiation doses in a specific DBT system with realistic 2D (the X‐ray tube is fixed) and 3D (the X‐ray tube is moved) image acquisitions for three models of breast thickness and density. We provided data regarding entrance surface air kerma (ESAK) and AGD derived by OSLD for comparison with an ionization chamber and the Monte Carlo simulation.

## 2 MATERIALS AND METHODS

The MAMMOMAT Inspiration system (Siemens Medical Solutions Inc., Erlangen, Germany) with dual acquisition modes for 2D and 3D imaging was used. The X‐ray tube is fixed in 2D acquisition mode, whereas it rotates for different tube angles between −25° and +25° at 2° intervals in 3D acquisition mode. In addition, a periodic quality control program was performed to ensure the reliability of the DBT system.

### 2.1 OSLD calibration

A nanoDot (Landauer Inc., Glenwood, IL, USA) is a small‐type of OSLD that has a disc of Al_2_O_3_:C enclosed in a plastic case that is 10 × 10 × 2 mm. It was used together with a microStar reader (Landauer Inc., Glenwood, IL, USA) to read out dose values for all measurements. The lower limit of detection of this OSLD system is 46.7 μGy, according to the calibration certificate provided by the manufacturer. The nanoDots were calibrated with a parallel‐plate ionization chamber (10 × 6‐6 M: Radcal Corp., Monrovia, CA, USA) to correct its energy response to the experimental conditions, as shown in Table 1, as well as the mass–energy absorption of nanoDots to air. The calibration factor of the nanoDots (C) for each experimental condition was determined by following formula:

(2) $$C=\frac{K_{i}}{K_{o}-\;\text{BG}},$$

where $K_{i}$ is the average incident air kerma from the ionization chamber (mGy), $K_{o}\;$is the average incident air kerma readout from the nanoDots (mGy), and BG is the average background readout from the control nanoDots (mGy).

**TABLE 1:** The exposure parameters were provided by the automatic exposure control in the DBT system, to determine the ESAK and the AGD

					Tube‐current time (mAs)	
PMMA thickness (cm)	Breast equivalent thickness (cm)	Tube voltage (kV)	HVL (mmAL)	Target/filter	2D mode	3D mode
2.0	2.1	25	0.52	W/Rh	71	71
4.0	4.5	28	0.54	W/Rh	125	110
7.0	9.0	34	0.59	W/Rh	200	180

### 2.2 Dose measurement and AGD determination

**FIGURE 1:**
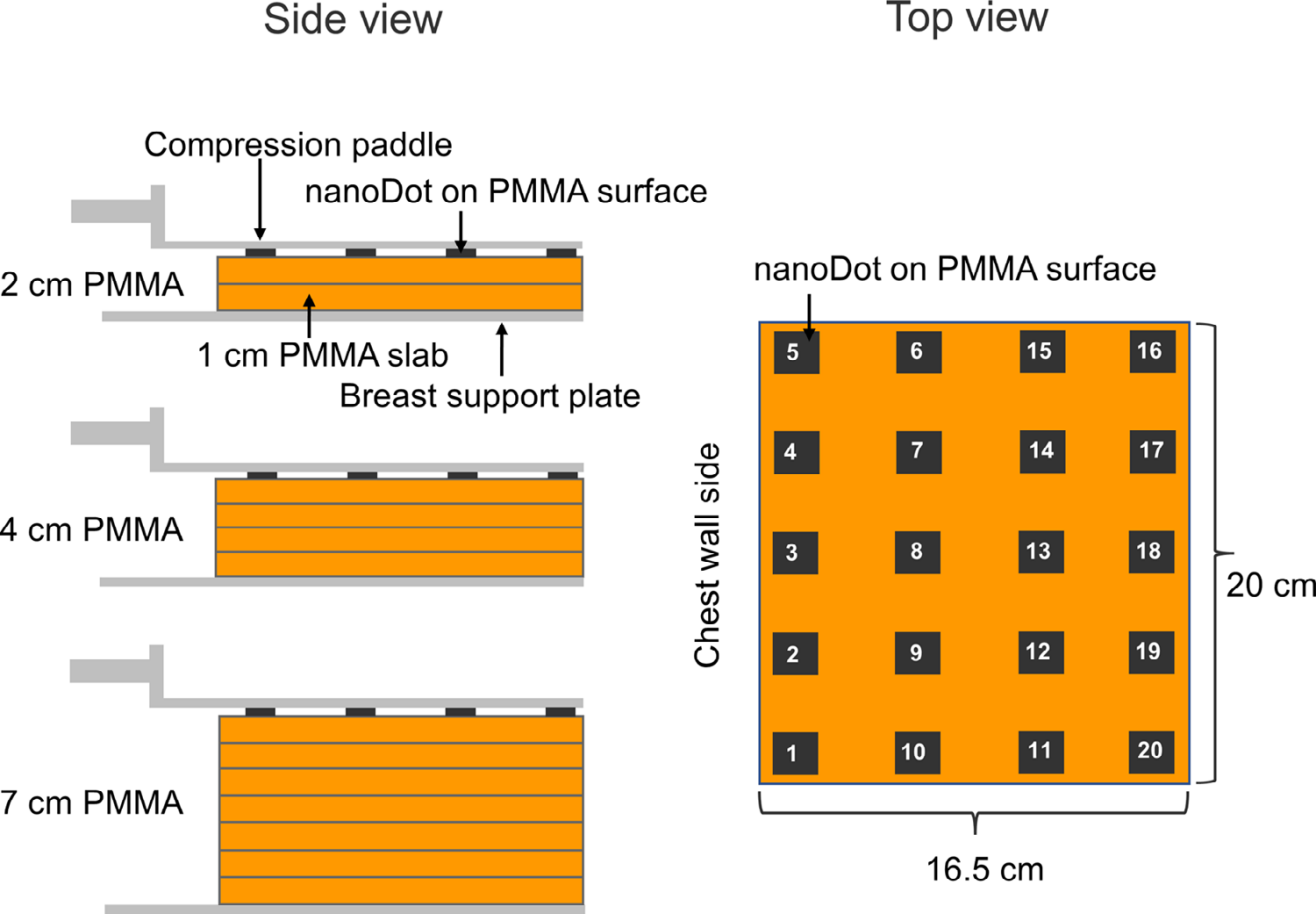
The experimental setup for the ESAK measurement using nanoDots

The polymethyl methacrylate (PMMA) thicknesses of 2.0, 4.0, and 7.0 cm were used as equivalent breast thickness of 2.1 cm (97% glandular tissue), 4.5 cm (40% glandular tissue), and 9.0 cm (4% glandular tissue), respectively, according to the protocol for the quality control of the physical and technical aspects of DBT systems.^4^ The equivalent PMMA thicknesses consisted of a set of 1 cm rectangular PMMA slabs, as shown in Figure 1. The exposure parameters for imaging a set of PMMA using 2D and 3D acquisition modes were automatically selected by an automatic exposure control in the DBT system, as shown in Table 1.

In this study, *K* was measured regarding the protocol for the quality control of the physical and technical aspects of the DBT systems using a parallel‐plate ionization chamber (IC) placed at 6 cm from the chest wall edge in contact with the compression paddle.^4^ The X‐ray tube was fixed at the zero degrees position for the K measurement with 2D and 3D acquisition modes according to the recommendations of the quality control protocol.^4^ The *K* measurements were performed with exposure parameters given in Table 1 for 2D and 3D acquisition modes. The AGD was determined for 2D and 3D modes by using the formula (1).

The ESAK was measured by nanoDots using the same set of PMMA thicknesses as mentioned above. The 20 nanodDots were used to determine the ESAK for the area of the compressed breast during mammography and to mimic the condition in the clinical setting. It is a surrogate of ESAK obtained by a patient in a real clinical setting. Twenty nanoDots were placed on the surface of the PMMA phantom to measure the ESAK, as shown in Figure 1. The PMMA set was irradiated three times at each exposure setting under 2D and 3D mode operations. The radiation doses were allowed to accumulate in the nanoDots for the three irradiations. Then, each nanoDot was read consecutively three times using a microStar reader, along with three nanoDots used as a control to reduce measurement uncertainty; the average of the three readings from each nanoDot was used for calculating the ESAK per irradiation.

The AGD was calculated using *K* measured by nanoDot from the 2D and 3D modes; the ESAK was obtained from the nanoDot placed 6 cm from the chest wall edge, as shown by the number 8 in Figure 1. The ESAK was subsequently divided by the backscatter factor (BSF) to calculate *K*, which was obtained from a Monte Carlo simulation in Section 2.3. The AGD was calculated for 2D and 3D modes by multiplying *K* with *g*, *c*, and *s* conversion factors, as given in Table 2.

**TABLE 2:** The calibration factors for nanoDots and conversion factors were used to calculate the AGD for the DBT

		AGD conversion factors			
kV	Calibration factors for nanoDots	g	c	s	T
25	1.053	0.540	0.914	1.042	0.979
28	1.005	0.306	1.035	1.042	0.970
34	1.021	0.169	1.251	1.042	0.962

### 2.3 Monte Carlo simulation

We used the geometry of the MAMMOMAT Inspiration system that was modeled by the Particle and Heavy Ion Transport Code System (PHITS) version 3.08 in our previous study.^13^ The MoXS‐3 simulation software was used for estimating the X‐ray energy spectrum of the original DBT source by using tube voltage, target angle, and the thickness of inherent filters.^14^ The W/Rh spectra of 25, 28, and 34 kV were calculated by the MoXS‐3 simulation software and used in the PHITS simulation to determine the ESAK for the DBT system. A nanoDot with an Al_2_O_3_:C disc inside was defined according to the instructions by Kerns et al.^15^ The nanoDot and PMMA composition data were obtained from the National Institute of Standards and Technology.^16^ A PMMA phantom with the 20 nanodots placements was similarly defined according to the experimental setup described in Section 2.2. The fixed X‐ray tube was defined for the 2D acquisition mode. The X‐ray tube rotated between −25° and +25°, whereas exposure was taken at every 2° for the 3D acquisition mode. The 10^6^ photons per batch for each projection were used to calculate ESAK in the simulation. The ESAK was calculated by multiplying the simulated output dose from each angle of the X‐ray tube rotation with the weight fraction for the number of projections. The weight fraction was 100% divided by 25 projections. Consequently, the total ESAK for the 3D mode was a summation of ESAK obtained from each angle of the X‐ray tube rotation. The heeling effect in the DBT simulation was considered by inserting a radiation field with a wedge filter in regard to the measured data. The low‐energy cutoff was set to 1 keV. The ESAK was calculated in Al_2_O_3_:C discs with the presence of PMMA phantom by using T‐deposit tally, and the BSF was calculated by the ratio of ESAK to K. The PMMA phantoms were removed to obtain K in the Al_2_O_3_:C discs that had been located on the PMMA surface. The ratio of the mass–energy absorption coefficient between the nanoDots and air was applied in the simulation for the dose calculation. The dose calculation was based on a statistical uncertainty of less than 2.0% according to the PHITS model definition.^17^ From the nanoDot numbered 8, the *K* was derived and used to calculate AGD with *g*, *c*, and *s* conversion factors, as given in Table 2.

## 3 RESULTS

**FIGURE 2:**
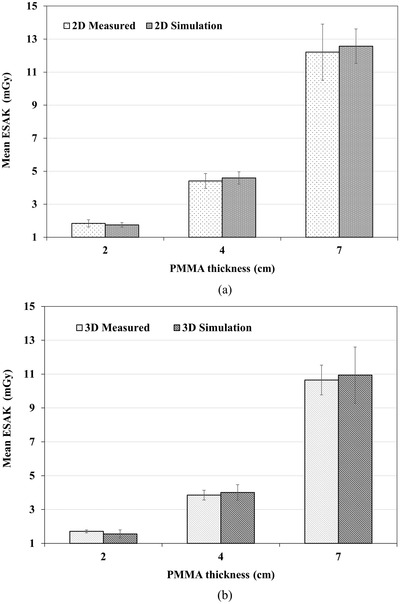
The mean of ESAK for the area of the compressed breast obtained by the 20 nanoDots from the measurement and simulation for 2D (a) and 3D modes (b)

**FIGURE 3:**
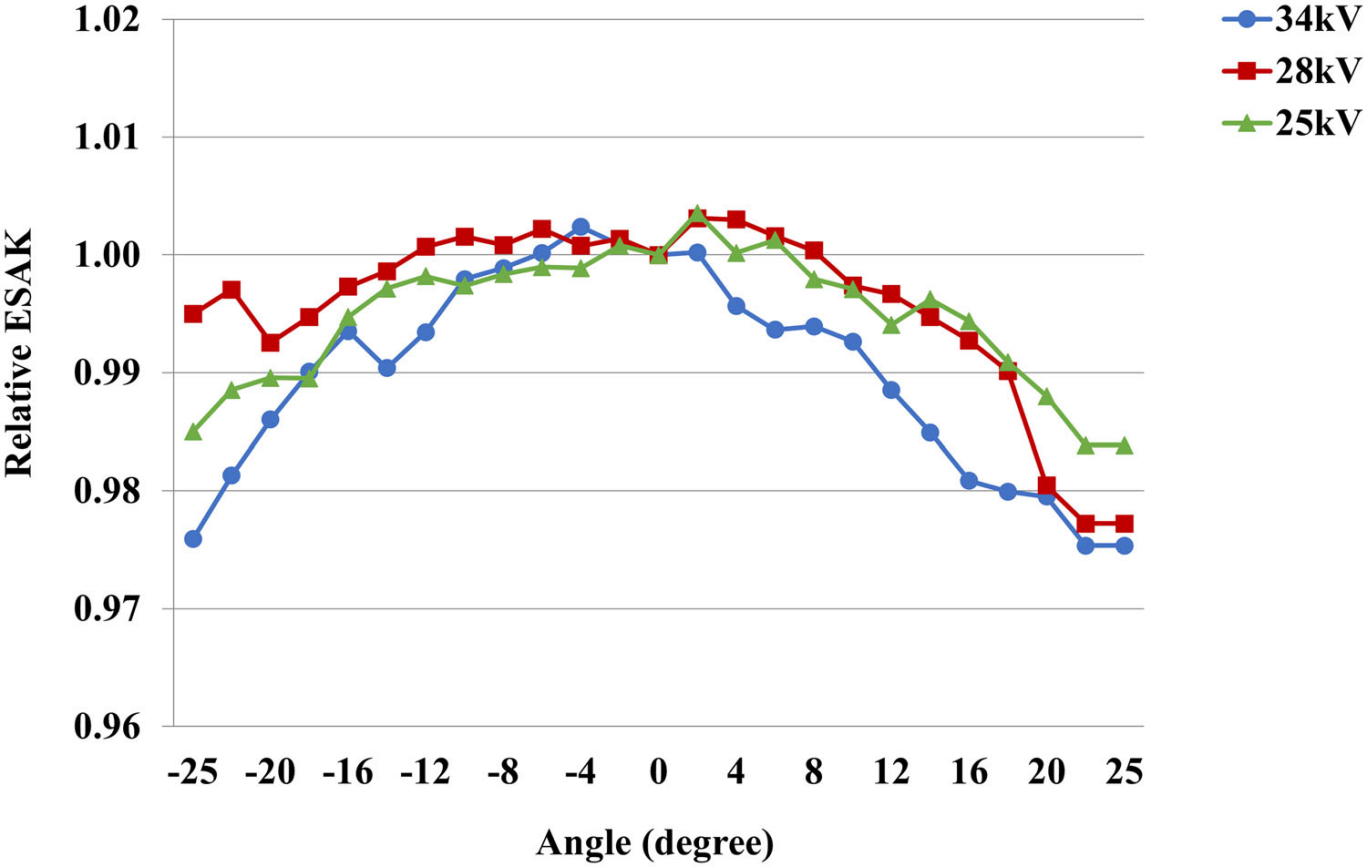
The nanoDots response with angles of irradiation between −25° and +25° calculated from simulation for the 3D acquisition mode with three phantom thicknesses corresponding to three tube voltages

The calibration factors for nanoDots when using an X‐ray beam of 25, 28, and 34 kV are presented in Table 2. The nanoDots responded to the kV range with an accuracy of ±5.0%. The results of the BSF in Table 3 were calculated from 2.0, 4.0, and 7.0 cm PMMA thickness using the Monte Carlo simulation. The BSF increased by increasing the PMMA thickness. The results of BSF showed a small difference of less than 1.0% between 2D and 3D modes. The results of the mean and standard deviation of ESAK obtained by the 20 nanoDots in which placed at the phantom surface (Figure 1) from the measurement and simulation are shown in Figure 2. It was found that the simulation overestimated the ESAK compared to the nanoDots measurement at the 28 and 34 kV techniques for both 2D and 3D modes. In contrast, the simulation underestimated the ESAK compared to the measurement of the 25 kV technique for both 2D and 3D modes. The difference of ESAK between the simulation and the measurement ranged from −8.8% (25 kV, 3D mode) to 4.2% (28 kV, 3D mode). The results showed that the variation arising from measuring the ESAK with nanoDots (8.6–13.2% coefficient of variance [CV]) varied largely compared to the simulation (6.0–8.3% CV) of both 2D and 3D modes. The variations of nanoDots response with angles of irradiation between −25° and +25° were calculated from simulation for the 3D acquisition mode with the three phantom thicknesses (2, 4, and 7 cm) corresponding to the tube voltages of 25, 28, and 34 kV. The relative ESAK for each irradiated angle was calculated by dividing the mean ESAK from 20 nanoDots of a certain irradiated angle with those of 0°. The results are shown in Figure 3. The relative ESAK values were slightly decreased at the irradiated angles beyond 0° by a maximum of 2.5% (±25°, 34 kV) compared to 0°. The nanoDots response decreased with the increase of irradiated angles. The results of AGD derived from nanoDots for comparison with the IC and the Monte Carlo simulation are shown in Table 4. Specifically, the AGD for the nanoDots and the simulation was calculated according to formula (1) by using the *K* and conversion factors (*g*, *c*, *s*) in Table 2. The best agreement with a deviation of less than ±2.3% was observed between the IC and the simulation from their determination of the AGD in both 2D and 3D modes. Except for comparing the simulation with IC in 3D mode, the AGD showed an unexpected discrepancy at 25 kV by −10.6%. The discrepancies were within ±4.3% for both 2D and 3D modes when comparing the nanoDots and the simulation, except −9.9% for the 3D mode at 25 kV. When comparing the nanoDots and the IC, the AGD discrepancies were within ±2.8% and ±4.2% for the 2D and 3D modes.

**TABLE 3:** The calculated backscatter factors from the Monte Carlo simulation

PMMA thickness (cm)	Backscatter factors	
	2D mode	3D mode
2.0	1.069	1.068
4.0	1.079	1.081
7.0	1.096	1.096

**TABLE 4:** The AGD was calculated using incident air kerma obtained from the IC, the nanoDot, and the Monte Carlo simulation

Breast thickness (cm)	AGD in 2D mode (mGy)			AGD in 3D mode (mGy)		
	IC	nanoDots	Simulation	IC	nanoDots	Simulation
2.1	0.91	0.94	0.92	0.89	0.88	0.80
4.5	1.50	1.51	1.52	1.28	1.25	1.29
9.0	2.71	2.66	2.77	2.35	2.45	2.35

## 4 DISCUSSION

The calculated BSF for W/Rh target/filter combination in this study showed consistency within ±3.9% of those reported as a function of half‐value layer (HVL) between 0.5 and 0.6 mm Al by the European Protocol on Dosimetry in Mammography.^18^ In comparison with a Monte Carlo simulation study by Kramer et al.,^19^ our results agree with the ±4.0% in regard to the 25, 28, and 34 kV for Rh/Rh target/filter combination and the calculation point on the phantom surface. These deviations were mainly contributed by the differences in BSF measurement points and monoenergetic versus polyenergetic beams. Specifically, this study calculated the BSF based on the central axis dose that averaged on the 16.5 cm × 20.0 cm phantom surface. The specific point at 6 cm from the chest wall edge with 1.0 cm × 1.0 cm on the phantom surface was performed in the previous study.^19^ In this study, the BSFs were averaged from 20 nanoDots placed in different locations. However, backscatter varies with the rotation angles of the X‐ray tube and locations of the nanoDots. The results from this study show that the BSF varies slightly with X‐ray energies, rotation angles of X‐ray tube, and locations of the nanoDots. Considering the angular dependence of the backscatter simulation for two places at central axis (the nanoDot number 8 in Figure 1) and off‐central axis (the nanoDot numbers 6 and 10 in Figure 1), the angular dependence averaged over all angles for the two places was less than 3.0% for the rotation angles of X‐ray tube in 3D mode. It was found that angular dependence increased with the increasing of tube voltages. The variations of BSF were lower than 1.5% in 2D mode and 3.0% in the 3D mode for the two places of nanoDots mentioned above. This is due to the difference in the locations of the nanoDots. The influence of the typical X‐ray beam difference on the BSF was a maximum of 5.0% as the polyenergetic beam gave a lower BSF compared to the monoenergetic beam in the Kramer et al. work.^19^ This confirms our results, that the BSF calculated for polyenergetic beam was lower than those of the monoenergetic beam in the Kramer et al. work.^19^ The angular dependencies of nanoDots were found to be less than 3.0% in our study. These agree with the lower of 4.0% in the study of Kawaguchi et al. It is because the nanoDots were fixed at 0° angular position, whereas X‐ray tube moved across them on the 3D acquisition mode for both our study and the study of Kawaguchi et al. In contrast, the nanoDots placement was rotated, whereas X‐ray tube was fixed at 0° angular position in the study of Alothmany et al.^11^

The calculated AGD for both the 2D and 3D modes by the IC measurement and the simulation had the best agreement with a variation of less than ±2.3%, except for the 3D mode at 25 kV (−10.6%). The errors on the ESAK determination by the simulation of the 25 kV technique subsequently influenced the AGD calculations, especially for the 3D mode. This may be due to the limitations of our simulation model that the attenuation of low‐energy X‐rays were overestimated, whereas the X‐ray tube moved from −25° to +25°. The discrepancies of the obtained AGD between the IC and nanoDots measurements were a maximum of 2.8% for the 2D mode and 4.2% for the 3D mode. It is considered the variation of the nanoDots response with beam energies and angles of irradiation. The 5.8% overall uncertainty of the nanoDot measurements (U) in this study could be obtained using the following formula:

(3) $$U=\sqrt{5.0{\%}^{2}+3.0{\%}^{2}},$$

where 5.0% was the beam energy response of a nanoDot and 3.0% was the variations of the nanoDots response with angles of irradiation. The overall uncertainty was lower than 10.0% of the relative expanded uncertainty (*k* = 2) of the TLD measurements for the AGD estimation, which is provided in the International Atomic Energy Agency technical reports series number 457 (TRS 457).^20^ Currently, the uncertainty of OSLD (nanoDot) measurements for the AGD estimation is not available in the TRS 457. The limitation of this study was the AGD estimation in one DBT system with a W/Rh target/filter combination and the X‐ray tube rotated in the range of ±25°. The AGD determination using the nanoDots with the varieties of target/filter combinations and the ranges of X‐ray tube rotation will give more information regarding their validity and reliability.

## 5 CONCLUSION

NanoDots were used to measure the ESAK using a fixed X‐ray tube for the 2D mode, and for the 3D mode, the X‐ray tube was rotated to determine AGD. When the sensitivity of nanoDots was corrected against the IC for each experimental condition, the AGD discrepancies fell within ±2.8% for the 2D mode and ±4.2% for the 3D mode, when compared with the standard protocol by IC. The results were similarly observed for the simulation, except for the 3D mode at 25 kV. The main factors, including the beam energy response and the angular dependence of the nanoDots, contributed to the AGD discrepancies. However, it was less than ±10.0% with regard to the relative expanded uncertainty of the TLD measurements for the AGD estimation according to the TRS 457. The nanoDots can be used as an alternative dosimeter on the dose measurement with realistic 2D and 3D image acquisitions in the specific DBT system for AGD determination without an additional T factor.

## CONFLICT OF INTEREST

No conflicts of interest
